# Efficacy and Safety of Nifurtimox in Pediatric Patients with Chagas Disease: Results at 4-Year Follow-Up in a Prospective, Historically Controlled Study (CHICO SECURE)

**DOI:** 10.1128/aac.01193-22

**Published:** 2023-03-28

**Authors:** Jaime Altcheh, Victor Sierra, Teresa Ramirez, Jimy José Pinto Rocha, Ulrike Grossmann, Erya Huang, Guillermo Moscatelli, Olivia Ding

**Affiliations:** a Parasitología, Hospital de Niños Ricardo Gutiérrez and Instituto Multidisciplinario de Investigacion en Patologias Pediatricas (IMIPP), CONICET-GCBA, Buenos Aires, Argentina; b Centro de Atención e Investigación Médica S.A., Yopal, Colombia; c Centro de Enfermedad de Chagas y Patologias Regionales, Santiago del Estero, Argentina; d Fundación CEADES–Plataforma de Atención Integral a los Pacientes con Enfermedades de Chagas, Cochabamba, Bolivia; e Bayer AG, Research and Development Pharmaceuticals, Berlin, Germany; f Bayer US LLC, Whippany, New Jersey, USA; g Bayer HealthCare Co., Ltd., Shanghai, China

**Keywords:** Chagas disease, *Trypanosoma cruzi*, nifurtimox, pediatrics, treatment, seronegative conversion, follow-up

## Abstract

Nifurtimox is recommended for the treatment of Chagas disease; however, long-term follow-up data are scarce. This prolonged follow-up phase of the prospective, historically controlled, CHICO clinical trial evaluated seronegative conversion in pediatric patients aged <18 years with Chagas disease who were followed for 4 years after nifurtimox treatment. Patients were randomly assigned 2:1 to nifurtimox 60-day or 30-day regimens comprising 10 to 20 mg/kg/day for patients aged <12 years and body weight <40 kg, and 8 to 10 mg/kg/day for those aged ≥12 years and body weight ≥40 kg. Anti-Trypanosoma cruzi antibodies decreased during the study period, achieving seronegative conversion in 16 (8.12%) and 8 (8.16%) patients in the 60-day and 30-day nifurtimox regimens, respectively, with corresponding incidence rates per 100 patients/year of seronegative conversion of 2.12 (95% confidence interval [CI]: 1.21 to 3.45) and 2.11 (95% CI: 0.91 to 4.16). Superiority of the 60-day nifurtimox regimen was confirmed by the lower limit of the 95% CI being higher than that (0%) in a historical placebo control group. Children aged <2 years at baseline were more likely to reach seronegative conversion during the 4-year follow-up than older children. At any annual follow-up visit, >90% of evaluable patients had persistently negative quantitative PCR results for T. cruzi DNA. No adverse events potentially related to treatment or caused by protocol-required procedures were documented for either treatment regimen. This study confirms the effectiveness and safety of a pediatric formulation of nifurtimox administered in an age- and weight-adjusted regimen for 60 days to treat children with Chagas disease.

## INTRODUCTION

Chagas disease (American trypanosomiasis) is a potentially life-threatening parasitic disease caused by the hemoflagellate protozoan Trypanosoma cruzi (1). Historically, the disease has predominantly affected people in South and Central America (https://www.cdc.gov/parasites/chagas/gen_info/detailed.html). However, the epidemiology of Chagas disease has changed in recent decades due to an increase in the migration of infected (but typically asymptomatic) individuals from rural areas to cities and from countries of endemicity to countries of non-endemicity (2, 3). Thus, recent reports estimate that, of approximately 8 million individuals infected with T. cruzi worldwide, most are resident in Latin America (3), but 300,000 to 500,000 infected individuals are living in North America and up to 120,000 cases have been reported in Europe (2).

Chagas disease has two phases: an initial acute phase of 6 to 8 weeks and then, if the patient is not treated, a chronic phase, which can be indeterminate, lasting years or decades, or symptomatic. About two-thirds of patients display the indeterminate chronic form without clinical signs or symptoms, but in up to 40% of patients, Chagas disease can progress to the symptomatic chronic form 10 to 30 years after the initial infection, with affected patients experiencing a range of clinical manifestations, including cardiac, digestive system, neurological, or combined disorders. The most serious manifestation of the disease is Chagasic cardiomyopathy (4, 5), which is a rapidly expanding health problem worldwide (2).

Two pharmacological agents have been available for the treatment of T. cruzi infection for more than 50 years: nifurtimox and benznidazole, both of which are effective antitrypanosomal drugs (6). A new formulation of nifurtimox allows the 30 mg and 120 mg dose strength tablets to be divisible and dispersible, which addresses the specific needs associated with the treatment of children who are not able to swallow tablets (7). Approved by the FDA in 2020 for the treatment of Chagas disease in children, nifurtimox is the only drug recommended for infants younger than 2 years of age with Chagas disease in the United States (https://www.accessdata.fda.gov/drugsatfda_docs/label/2020/213464s000lbl.pdf).

The current standard criterion for cure involves the conversion of serological response to negative as measured by at least two different types of conventional serological assay, such as enzyme-linked immunosorbent assay (ELISA), indirect hemagglutination assay (IHA), or indirect immunofluorescence assay (8–10). However, the strong antibody response of the T. cruzi parasite, which persists even after successful antitrypanosomal treatment and elimination of the parasite, makes establishing the success of a particular treatment challenging in clinical studies (11). In chronic Chagas disease, it may take approximately 10 to 20 years of posttreatment follow-up to achieve seronegative conversion (12–15). A decrease in anti-T. cruzi serological reactivity, combined with persistently negative parasitological tests, may indicate a response to treatment (13, 16, 17).

We evaluated the changes in serological profile in pediatric patients with acute or chronic Chagas disease following treatment with a new formulation of nifurtimox for 60 days. Patients were enrolled in a prospective, historically controlled, phase III clinical trial: the **CH**agas disease **I**n **C**hildren treated with nifurtim**O**x follow-up for **SE**roconversion and **CURE** (CHICO SECURE) trial. The results of the first year of posttreatment follow-up in the CHICO trial have been reported previously (18). Patients were then followed for a further 3 years, resulting in a total posttreatment follow-up period of 4 years. The incidence rate of seronegative conversion of anti-T. cruzi antibodies, measured and confirmed by conventional recombinant ELISA and IHA, observed for nifurtimox was compared with that in historical controls who received placebo. The effect of nifurtimox treatment on T. cruzi parasitemia, measured by quantitative PCR (qPCR) assay, was also evaluated during the study and is reported here, together with the safety findings during follow-up.

## RESULTS

### Patient demographics and characteristics.

Of the 330 pediatric patients randomized and treated with nifurtimox in CHICO study (18), a total of 295 pediatric patients were enrolled at 17 sites in Argentina (*n *= 161), three sites in Bolivia (*n *= 54), and three sites in Colombia (*n *= 80) and followed for 4 years posttreatment. The demographic and patient characteristics of enrolled patients are summarized in Table 1. Overall, 53.2% were female, and the patients' mean body mass index was 18.94 kg/m^2^. The median age was 8.5 years (interquartile range: 2 to 13 years). At randomization, one-third of the patients were under the age of 7 years (29.2%), including 17 children younger than 8 months (5.8%); one-third were aged between 7 and 12 years (37.3%); and one-third were aged between 13 and 17 years (33.6%). All patients enrolled were diagnosed with Chagas disease according to the inclusion criteria defined in the CHICO study (18).

**TABLE 1:** Demographic and patient characteristics at baseline (full analysis set)^*a*^

Characteristic	Nifurtimox regimen		Total (*N *= 295)
	60-day (*n *= 197)	30-day (*n *= 98)	
Sex, *n* (%)			
Male	89 (45.2)	49 (50)	138 (46.8)
Female	108 (54.8)	49 (50)	157 (53.2)
Age group, *n* (%)			
≤2 yrs	30 (15.2)	13 (13.3)	43 (14.6)
3 to ≤6 yrs	31 (15.7)	12 (12.2)	43 (14.6)
7 to ≤12 yrs	73 (37.1)	37 (37.8)	110 (37.3)
13 to <18 yrs	63 (32.0)	36 (36.7)	99 (33.6)
BMI, mean (SD), kg/m^2^	18.68 (3.99)	19.46 (4.02)	18.94 (4.01)
Total purified antigen ELISA test, *n* (%)			
Reactive	197 (100)	97 (99)	294 (99.7)
Nonreactive	0	1 (1)	1 (0.3)
IHA test, *n* (%)			
Reactive	194 (98.5)	98 (100)	292 (99)
Nonreactive	2 (1)	0	2 (0.7)
Missing	1 (0.5)	0	1 (0.3)
Recombinant ELISA test, *n* (%)			
Reactive	197 (100)	97 (99)	294 (99.7)
Nonreactive	0	1 (1)	1 (0.3)
Quantitative PCR test, *n* (%)			
Detectable	105 (53.3)	48 (49)	153 (51.9)
Not detectable	89 (45.2)	49 (50)	138 (46.8)
Nonevaluable	1 (0.5)	1 (1)	2 (0.7)
Missing	2 (1)	0	2 (0.7)

a Age was defined as the patient's age at the time of randomization in CHICO (18). BMI, body mass index; ELISA, enzyme-linked immunosorbent assay; IHA, indirect hemagglutination assay.

### Duration of observation.

The median duration of observation, defined as the actual number of years an individual was at risk during the study, was 4.0 years for patients in both treatment groups (Table 2). For the majority of patients (86.8%), the duration of observation was 3 to 4 years. Of note, the first year of posttreatment follow-up observation was included in CHICO (18).

**TABLE 2:** Duration of observation (full analysis set)

Duration of observation (yrs)^*a*^	60-day nifurtimox regimen (*n *= 197)	30-day nifurtimox regimen (*n *= 98)	Total (*N *= 295)
0^*b*^−1, *n* (%)	8 (4.1)	4 (4.1)	12 (4.1)
1–2, *n* (%)	5 (2.5)	2 (2.0)	7 (2.4)
2–3, *n* (%)	5 (2.5)	3 (3.1)	8 (2.7)
3–4, *n* (%)	173 (87.8)	83 (84.7)	256 (86.8)
4–5, *n* (%)	6 (3.0)	6 (6.1)	12 (4.1)
Mean (SD)	3.83 (0.73)	3.87 (0.73)	3.84 (0.73)
Median (IQR^*c*^)	4.00 (4)	4.00 (4)	4.00 (4)

a Duration of observation was the actual number of years a patient was at risk in the study, i.e., the time from when a patient was randomized in the study until they were confirmed as having seroconversion, withdrawn from the study, used antitrypanosomal therapy, or completed the study, whichever was the earliest. The duration of observation has been rounded to the nearest integer.

b One patient initiated treatment with benznidazole <0.5 years after randomization.

c IQR, interquartile range.

### Seronegative conversion.

During the 4-year posttreatment follow-up, seronegative conversion was observed in a total of 16 (8.12%) and 8 (8.16%) patients in the 60-day and 30-day nifurtimox regimens, respectively (Table 3). The incidence rate of seronegative conversion was 2.12 100 patients/year (95% confidence interval [CI]: 1.21–3.45 100 patients/year) in the 60-day treatment group and 2.11 100 patients/year (95% CI: 0.91–4.16 100 patients/year) in the 30-day treatment group. Superiority of the 60-day nifurtimox regimen compared with the historical placebo control (19) was confirmed by the lower limit of the 95% CI being above 0%.

**TABLE 3:** Number and incidence rate of seronegative conversion with nifurtimox treatment (full analysis set)

Parameter	60-day nifurtimox regimen (*n *= 197)	30-day nifurtimox regimen (*n *= 98)
Patients with seronegative conversion, *n* (%)	16 (8.12)	8 (8.16)
Total duration of observation, yrs^*a*^	754	379
Incidence rate^*b*^ (95% CI^*c*^), 100 patients/yr	2.12 (1.21–3.45)	2.11 (0.91–4.16)

a Total duration of observation is the estimated actual number of years at risk for all patients who contributed to the study.

b The incidence rate was the number of new cases of seronegative conversion during the study period (i.e., 4 years after the end of nifurtimox treatment) divided by the patient-time at risk. It was modeled using a Poisson distribution with a two-sided 95% exact CI.

c CI, confidence interval.

**FIG 1:**
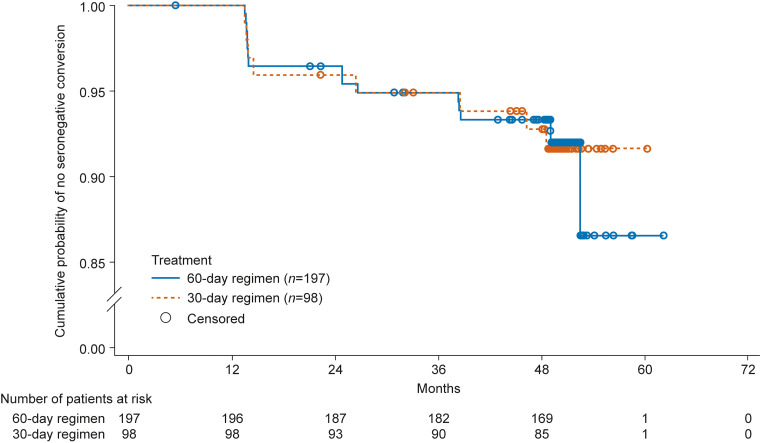
Kaplan–Meier curves of seronegative conversion in patients receiving 60-day or 30-day nifurtimox treatment regimens (full analysis set, *N *= 295). Patients who received other antitrypanosomal treatments were considered censored. Serological responses were measured by recombinant enzyme-linked immunosorbent assay and indirect hemagglutination assay, and negative results for both tests were required for the patient to be considered to have achieved seronegative conversion.

Overall, the number of new cases with seronegative conversion increased during the posttreatment follow-up. In the 60-day regimen, seronegative conversion, measured and confirmed by two types of assays (recombinant ELISA and IHA), was observed for the first time in 7 (3.55%), 3 (1.52%), 3 (1.52%), and 3 (1.52%) patients at the 1-, 2-, 3-, and 4-year posttreatment follow-up, respectively. The first observation of seronegative conversion in the 30-day nifurtimox regimen group at the corresponding follow-up time points was documented in 4 (4.08%), 1 (1.02%), 2 (2.04%), and 1 (1.02%) patients, respectively (Fig. 1). At the end of the study, the seronegative conversion rate was 7.11% in the 60-day nifurtimox regimen and 6.12% in the 30-day regimen.

In an exploratory analysis of serological response by age group, the highest number of seronegative conversions occurred in those aged 2 years or younger. In this age group, seronegative conversion was observed in 13 of 30 patients (43.33%) in the 60-day regimen and 7 of 13 patients (53.85%) in the 30-day regimen, with corresponding incidence rates of 14.61 100 patients/year (95% CI: 7.78–24.98 100 patients/year) and 18.92 100 patients/year (95% CI: 7.61–38.98 100 patients/year), respectively (Table 4). The incidence rate of all patients in the remaining age groups treated with the 60- or 30-day nifurtimox regimen was less than 1%. At the 4-year follow-up in the 60-day regimen, seronegative conversion was observed in one of 31 patients (3.23%) aged >2 to 6 years and in two of 73 patients (2.74%) aged >6 to 12 years. For patients older than 2 years and treated with nifurtimox for 30 days, only one patient seroconverted to negative (Table 4).

**TABLE 4:** Seronegative conversion measured by two types of assay (recombinant ELISA and IHA) according to age 4 years after the end of nifurtimox treatment (full analysis set)

Parameter	≤2 yrs (*N *= 43)		>2 to ≤6 yrs (*N *= 43)		>6 to ≤12 yrs (*N *= 110)		>12 to <18 yrs (*N *= 99)	
Nifurtimox regimen, d	60	30	60	30	60	30	60	30
Patients, *n*	30	13	31	12	73	37	63	36
Patients with seronegative conversion, *n* (%)	13 (43.33)	7 (53.85)	1 (3.23)	0	2 (2.74)	1 (2.70)	0	0
Total duration of observation, yrs^*a*^	89	37	122	48	291	151	252	143
Incidence rate (95% CI), 100 patients/yr^*b*^	14.61 (7.78, 24.98)	18.92 (7.61, 38.98)	0.82 (0.02, 4.57)	0	0.69 (0.08, 2.48)	0.66 (0.02, 3.69)	0	0

a Total duration of observation is the estimated actual number of years at risk for all patients who contributed to the study.

b The incidence rate was the number of new cases of seronegative conversion during the study period (i.e., 4 years after the end of nifurtimox treatment) divided by the patient-time at risk. It was modeled using a Poisson distribution with a two-sided 95% exact CI. Negative results for both recombinant enzyme-linked immunosorbent assay (ELISA) and indirect hemagglutination assay (IHA) were required for the patient to be considered to have achieved seronegative conversion.

### Seroreduction.

**FIG 2:**
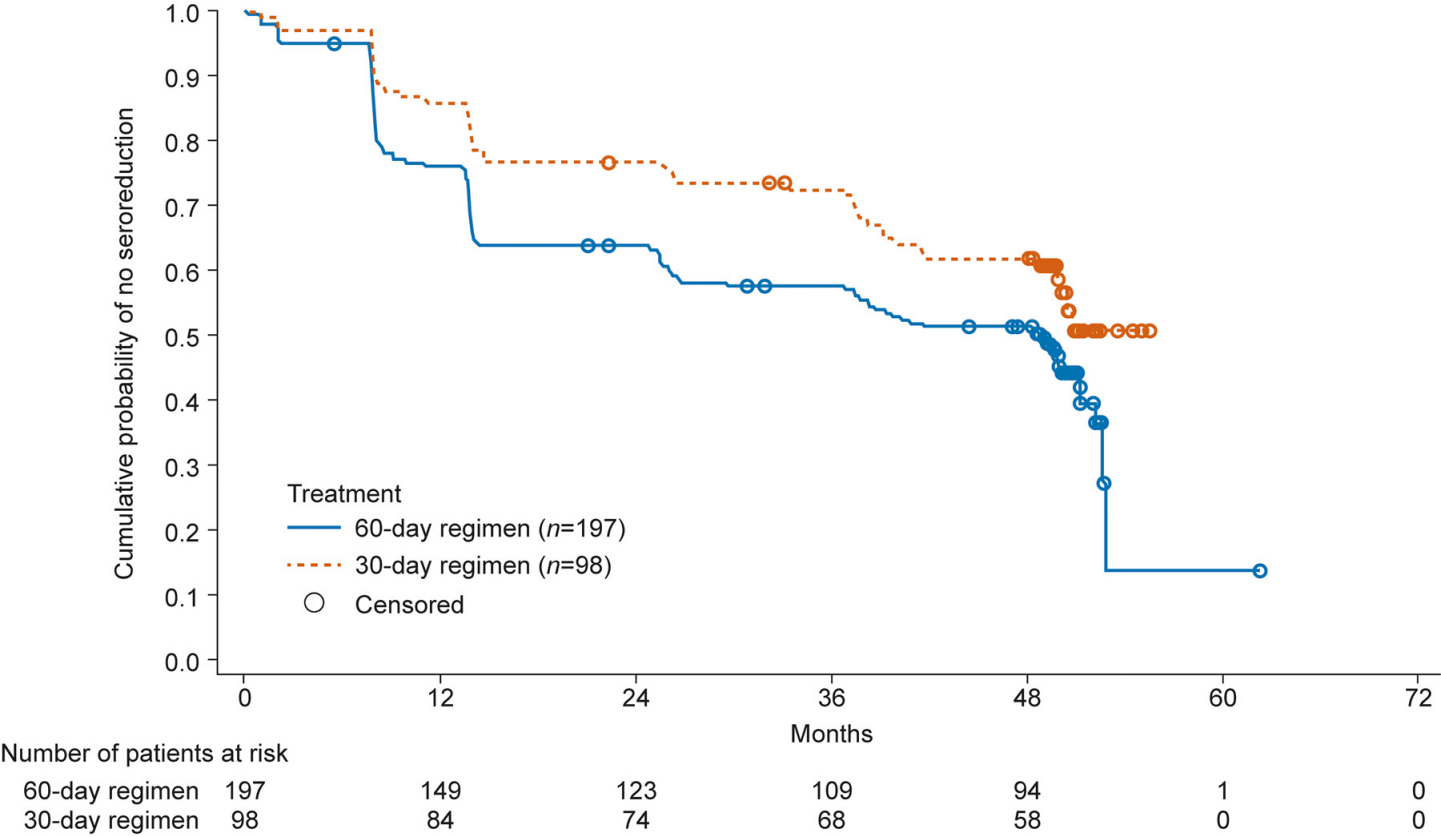
Kaplan–Meier curves of ≥20% to 100% seroreduction in patients receiving 60-day or 30-day nifurtimox treatment regimens (full analysis set, *N *= 295). Patients who received other antitrypanosomal treatments were considered censored. Serological responses were measured by reduction in optical density in two conventional enzyme-linked immunosorbent assay tests (recombinant ELISA and total purified antigen ELISA).

**FIG 3 F3:**
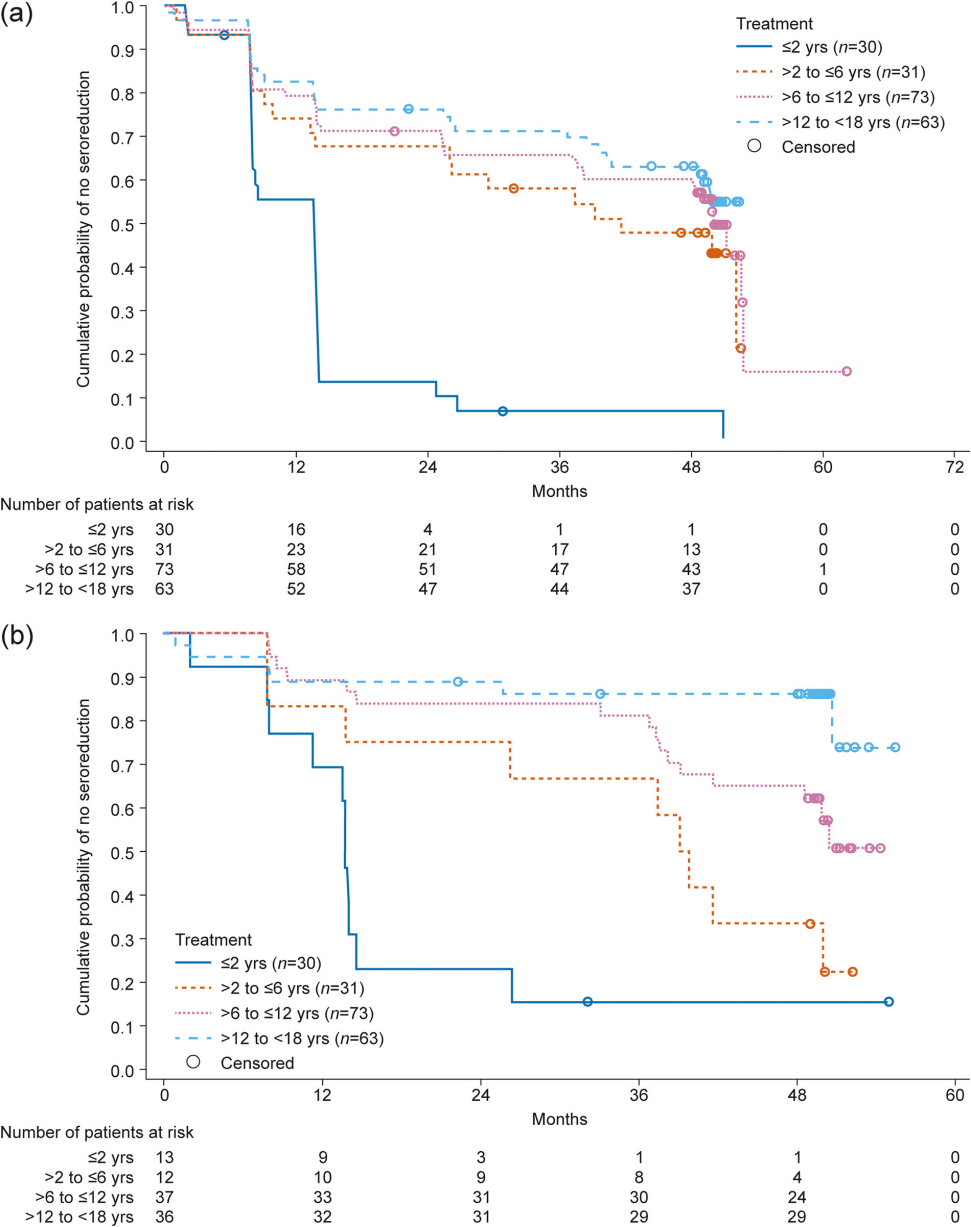
Kaplan–Meier curves of ≥20% to 100% seroreduction by age in patients receiving (a) 60-day and (b) 30-day nifurtimox treatment regimens (full analysis set, *N *= 295). Patients who received other antitrypanosomal treatments were considered censored. Serological responses were measured by reduction in optical density in two conventional enzyme-linked immunosorbent assay tests (recombinant ELISA and total purified antigen ELISA).

Optical density (OD) values measured by conventional ELISAs decreased over time in both treatment regimens during the study. At the 4-year posttreatment follow-up, the reduction in mean OD values from baseline was 23.72% and 18.68% by recombinant ELISA, and 23.81% and 19.08% by total purified antigen ELISA in the 60-day and 30-day treatment regimens, respectively. In Kaplan–Meier analyses that generated probability estimates for ≥20% to 100% seroreduction in OD values from baseline measured by two conventional ELISAs (recombinant ELISA and total purified antigen ELISA), a progressive reduction was seen in both treatment groups. At the 4-year follow-up, ≥20% to 100% seroreduction of around 50% and 40% was observed in the 60- and 30-day treatment regimen groups, respectively (Fig. 2). The probability of seroreduction was greater in younger patients in both treatment groups (Fig. 3).[Fig F3]

### T. cruzi DNA detection by qPCR.

More than 90% of patients in both treatment groups showed successive negative T. cruzi qPCR results at the annual posttreatment follow-up visits. A summary of the parasitological response measured by qPCR is shown in Table 5. Positive qPCR results were observed for only eight patients at any follow-up visit, with no clear differences in the pattern of occurrence between treatment groups. Six of these positive cases were observed at only one of the follow-up visits, while two patients tested positive at the end of the study. For one of these patients, ELISA values at the 4-year follow-up visit were missing, but qPCR results were also positive at the 3-year follow-up visit, when the mean conventional ELISA OD value had decreased from baseline by approximately 7%. For the other patient who was qPCR-positive at the 4-year follow-up visit, the change from baseline in mean conventional ELISA OD value was −2%.

**TABLE 5:** Parasitological response of patients according to follow-up visit (full analysis set), measured by quantitative PCR^*a*^

		Nifurtimox regimen		
Visit	T. cruzi qPCR	60-day (*n *= 197)	30-day (*n *= 98)	Total (*N *= 295)
Baseline	*n* Detectable Not detectable Nonevaluable Missing	197 (100) 105 (53.3) 89 (45.18) 1 (0.51) 2 (1.02)	98 (100) 48 (48.98) 49 (50.0) 1 (1.02) 0	295 (100) 153 (51.86) 138 (46.78) 2 (0.68) 2 (0.68)
1 yr posttreatment	*n* Detectable Not detectable Nonevaluable Missing	194 (98.48) 1 (0.51) 193 (97.97) 0 0	98 (100) 2 (2.04) 96 (97.96) 0 0	292 (98.98) 3 (1.02) 289 (97.97) 0 0
2 yrs posttreatment	*n* Detectable Not detectable Nonevaluable Missing	188 (95.43) 2 (1.02) 186 (94.42) 0 0	92 (93.88) 2 (2.04) 90 (91.84) 0 0	280 (94.92) 4 (1.36) 276 (93.56) 0 0
3 yrs posttreatment	*n* Detectable Not detectable Nonevaluable Missing	175 (88.83) 1 (0.51) 174 (88.32) 0 0	85 (86.73) 1 (1.02) 84 (85.71) 0 0	260 (88.14) 2 (0.68) 258 (87.46) 0 0
4 yrs posttreatment	*n* Detectable Not detectable Nonevaluable Missing	191 (96.95) 1 (0.51) 189 (95.94) 0 1 (0.51)	91 (92.86) 1 (1.02) 90 (91.84) 0 0	282 (95.59) 2 (0.68) 279 (94.58) 0 1 (0.34)

a Data shown are *n* (%).

### Serological response and Chagas disease-related cardiomyopathy.

No patients showed signs of established Chagas disease-related cardiomyopathy as measured by electrocardiogram (ECG) in the 60- or 30-day treatment groups at any of the posttreatment follow-up visits.

### Safety.

The safety of the nifurtimox regimens during treatment and at the 1-year follow-up has been reported previously (18). No adverse events (AEs) considered at least possibly related to nifurtimox, or AEs caused by protocol-required procedures as assessed by the investigator, were reported for patients in either treatment group during the subsequent 3 years of follow-up.

## DISCUSSION

Establishing the efficacy of antitrypanosomal therapy in patients with Chagas disease using the currently accepted criterion of treatment response is challenging. In Chagas disease, it is widely acknowledged that treatment success is difficult to measure with the current diagnostic tools because the parasites are highly antigenic and produce a strong antibody response that persists even after successful antiparasitic treatment (20). It can be many years after treatment before patients with chronic Chagas disease, particularly those who have been infected for a long time, become seronegative as assessed by conventional serological methods (21). For example, in one study of T. cruzi-infected children, most of whom had chronic indeterminate-stage disease, only 5 of the 29 children (17.2%) followed up at 2 years after trypanocidal treatment presented negative serology indicative of definitive cure (22). Because the currently accepted biomarker for cure—seronegative conversion—precludes the assessment of treatment response unless patients are followed for a prolonged time after treatment, parasitological tests (e.g., real-time PCR) are sometimes used for monitoring treatment response in patients with Chagas disease (23–26). However, all of these tests were initially developed as diagnostic tools and have not been validated for posttreatment follow-up (17). Long-term posttreatment follow-up studies in patients with Chagas disease are uncommon (27); those that have evaluated nifurtimox in children are rare and have typically been conducted in small groups of patients or individual cases (16).

In this study, we evaluated seronegative conversion in pediatric patients with Chagas disease followed over a period of 4 years posttreatment. The results confirm the effectiveness and safety of nifurtimox administered over 60 days in pediatric patients, from newborn to 17 years of age, with Chagas disease. The superior seronegative conversion attributable to the 60-day nifurtimox treatment regimen compared with historical placebo control that was demonstrated at 12 months after the end of treatment (18) was confirmed after a further 3 years of follow-up. The highest incidence rate of seronegative conversion in the 60-day treatment regimen was observed in pediatric patients aged 2 years or younger at baseline. This observation is consistent with the known characteristic in Chagas disease that effectiveness of antitrypanosomal therapy is higher if administered as soon as possible after infection and appears to decrease in proportion to the duration of infection. Moscatelli et al. (2019) report that children treated with benznidazole (*n *= 107) showed a progressive reduction in T. cruzi antibody titers measured by conventional ELISA and IHA (17). More than half of patients younger than 2 years achieved seronegative conversion at 36 months of follow-up. In children older than 2 years, a median (range) time to seronegative conversion was 72 (32–90) months; however, a persistent decrease in antibody titers was seen during the 3-year observation period (17) as was also observed in our study. An inverse relationship between age and seronegative conversion has similarly been shown for children treated with nifurtimox, with younger patients, particularly those up to 2 years of age, showing an earlier response and a higher probability of seroconversion after treatment (16). A recently reported retrospective cohort study of children treated with nifurtimox or benznidazole during childhood also demonstrated a significantly higher probability of seroconversion with younger age during a subsequent mean follow-up of 25 years (28). The results of these studies and the present study reinforce the importance of early diagnosis and prompt effective treatment of children who have acquired T. cruzi infection.

Conversion of serological response to negative measured by conventional serological testing remains the gold standard for monitoring the posttherapeutic response in Chagas disease (29). However, as noted, this approach is limited by the persistence of antibody titers, especially in chronic Chagas disease (19, 30). In our study a decrease in antibody titers seems to precede complete reversion of serological response to negative and may indicate an evolution toward cure from a clinical point of view. In the absence of biomarkers for evaluating early responses to antitrypanosomal therapy, especially in chronic Chagas disease, the clearance of parasitemia shown by persistently negative qPCR tests is sometimes used (8, 16, 17). The merits of qPCR as a rapid method for detecting the T. cruzi parasite are its speed, high specificity, and—at least in the acute phase of infection—sensitivity (31–33). These characteristics have favored the use of qPCR to monitor therapeutic responses in recent clinical trials of antiparasitic treatments (23–26). A limitation of this use of qPCR is the short duration of follow-up in such clinical trials in patients with established chronic infections (34). Furthermore, in the chronic phase of Chagas disease, PCR performs less well due to low and fluctuating parasitemia. A single negative qPCR result does not, however, exclude the presence of parasites in tissues or circulating in levels below the limit of detection (25), and a high proportion of patients with chronic infection demonstrated by conventional methods (20 to 60%) have a negative PCR result from tests on *ex vivo* blood sampled at a single time point (35). PCR/qPCR is thus not adequate to detect cure after antitrypanosomal therapy; however, a positive PCR/qPCR result is a useful marker of treatment failure (34, 36), making it an appropriate complement to serological testing for the follow-up of patients with chronic Chagas disease treated with antitrypanosomal therapy (8, 9, 14, 25). In our study, qPCR tests were persistently negative in most patients throughout follow-up. Positive qPCR results were observed in only eight patients at any follow-up visit and in two patients at the end of the study. It seems that in patients with a positive qPCR result at baseline, seroconversion to negative measured by conventional serology was observed more often compared with patients with a negative qPCR at baseline (data on file, Bayer AG). However, interpretation of the data is limited by the low number of cases observed. Nevertheless, the persistently negative successive qPCR tests that were observed reflect no reinfection, a low risk of therapeutic failure and a sustained beneficial effect of nifurtimox over the 4-year period of follow-up.

The previously reported results of the first year of posttreatment follow-up in this prospective trial (18) showed that the nifurtimox treatment regimen given for 30 days was not as effective as the 60-day regimen that was subsequently approved for use in the United States (https://www.accessdata.fda.gov/drugsatfda_docs/label/2020/213464s000lbl.pdf). In this prolonged posttreatment follow-up study, we continued to explore the outcomes of treatment with the shorter regimen using serological as well as parasitological tests. Interestingly, the incidence rate of seronegative conversion observed with the 30-day treatment regimen appears to be similar to that of the 60-day treatment regimen. Additionally, the majority of patients treated with nifurtimox for 30 days showed consecutively negative qPCR results during the posttreatment follow-up. However, the results need to be interpreted with caution as the study was not designed or powered to compare the two treatment regimens and therefore further studies are needed to confirm this observation.

Up to two-thirds of people with Chagas disease never develop signs or symptoms, even during the chronic infection phase (1, 37). The remaining 30% may eventually progress to develop severe cardiac and/or digestive manifestations (37), but predicting which patients will progress is an ongoing challenge. No patients, whether or not they seroconverted to negative during the study, showed signs of Chagas disease-related cardiomyopathy as assessed by ECG. Demonstrating the anticipated benefit of effective treatment of Chagas disease in childhood on the potential development of disease-related cardiomyopathy will require longer follow-up from larger studies or patient registries.

No AEs considered at least possibly related to nifurtimox treatment or caused by procedures required by the protocol were reported during the further 3 years of observation that followed CHICO (18). This reinforces the results of previous clinical and real-world studies that have reported the safety of nifurtimox in pediatric patients with Chagas disease (10, 18, 38, 39). Most treatment-related AEs occur within the first month of nifurtimox treatment (10). AEs arising from the regimens used in this study are thus likely to have been captured in the preceding 1-year study and were not anticipated in years 2–4 after treatment. Despite this and given the cellular effects (40) and experimental toxicity of nifurtimox in animals (41), we considered comprehensive monitoring of posttreatment safety to be an indispensable part of this study in young patients.

The main limitation of this study is the lack of a prospective untreated control group, which would not have been ethical to include because the current standard of care is the treatment of all infected children. The primary outcomes of treatment were compared with an estimated 0% seronegative conversion rate from historical placebo data obtained from a previously published clinical trial in 6- to 12-year-old children with early chronic Chagas disease treated with benznidazole and followed for 4 years (19). Although that study was a randomized, double-blind, placebo-controlled trial (19), it is not possible to take into account biases resulting from extrapolation of seroreactivity in a pediatric patient population that differs in age, time, and setting of diagnosis and treatment (18).

### Conclusions.

This study confirms the efficacy and safety of a pediatric formulation of nifurtimox administered in an age- and weight-adjusted regimen for 60 days to treat Chagas disease in pediatric patients with 4-year follow-up data. Children younger than 2 years treated with nifurtimox are more likely to show seronegative conversion than older children during a relatively short posttreatment follow-up observation. Reduction of ELISA OD values and consecutive negative T. cruzi qPCR results over time may indicate a therapeutic response and potential evolution toward serological cure. Further studies are needed to confirm a possible benefit of the 30-day nifurtimox treatment regimen. This 4-year posttreatment follow-up study extends the known positive benefit–risk ratio of nifurtimox in pediatric patients with Chagas disease.

## MATERIALS AND METHODS

### Ethics statement.

Approval from the ethics committee was obtained for all participating research sites, as described above (18). Written informed consent and/or permission from the participant and/or legally authorized parent(s) or representative(s) was obtained prior to selection for this study in accordance with the participant's age and local country regulations.

### Study design.

**FIG 4:**
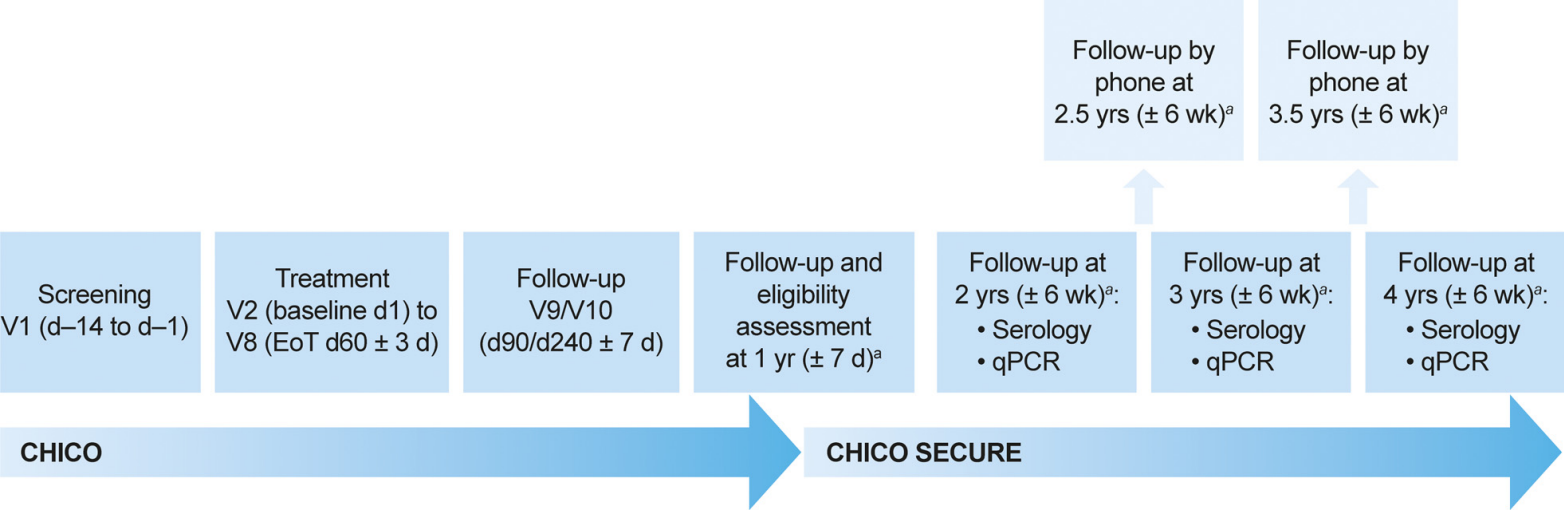
Study design and schedule of follow-up visits. The schedule of study visits V1 to V10 are as previously reported (18). ^a^The year of each follow-up visit was relative to EoT at d60. CHICO, **CH**agas disease **I**n **C**hildren treated with nifurtim**O**x; CHICO SECURE, **CH**agas disease **I**n **C**hildren treated with nifurtim**O**x – **SE**roconversion and **CURE**; d, day; EoT, end of treatment; qPCR, quantitative PCR; V, visit.

This was a prospective, randomized, blinded, historically controlled, parallel-group, long-term follow-up study (ClinicalTrials.gov identifier: NCT02625974) (Fig. 4). The study design, patients, inclusion/exclusion criteria, methods, and efficacy and safety results at 12 months posttreatment have been published previously (18). Briefly, boys and girls from newborn to <18 years of age with a confirmed diagnosis of Chagas disease were randomly assigned 2:1 to nifurtimox 60-day or 30-day treatment regimens. Nifurtimox was administered at 10 to 20 mg/kg/day for patients <12 years of age and body weight <40 kg, and 8 to 10 mg/kg/day for patients ≥12 years of age and body weight ≥40 kg, in three divided age- and weight-adjusted doses per day with food. This follow-up study was designed to evaluate seronegative conversion in patients who were followed for 4 years after the end of treatment with nifurtimox. The entire study (CHICO and CHICO SECURE parts) took place between January 2016 and August 2021, and CHICO SECURE was conducted between September 2018 and August 2021 at 23 research sites in three countries (Argentina, Bolivia, and Colombia) (see supplemental material).

### Study participants.

This study included patients who were randomized to treatment and received at least one dose of their assigned treatment with nifurtimox. Prior to enrollment, written informed consent was obtained from the patient and/or their parents or legally authorized representative(s) according to age and local regulations. Depending on their age, patient assent was also obtained if required by locally applicable laws and regulations in each country. Patients were considered to have completed the study if they completed the 4-year posttreatment follow-up (Fig. 4).

### Outcome measures.

The primary outcome was the incidence rate of seronegative conversion measured and confirmed by recombinant ELISA (42) and IHA in patients who were randomized and received at least one dose of nifurtimox in the 60-day treatment regimen. Verification of seroconversion required negative results for both tests. The incidence rate was calculated as the number of new cases of seronegative conversion during the study period (i.e., 4 years after the end of nifurtimox treatment) divided by the patient-time at risk. The duration of observation was the actual number of years a patient was at risk in the study, i.e., the time from when a patient was randomized in the study until he or she was confirmed as having negative seroconversion, withdrew from the study, used antitrypanosomal therapy, or completed the study, whichever was the earliest. Any patients who received other antitrypanosomal drugs (e.g., benznidazole) during the study were not included in the numerator for the calculation of seronegative conversion rate, but the period of observation prior to antitrypanosomal treatment was included in the calculation of cumulative patient-time.

Secondary outcomes investigated included: the incidence rate of seronegative conversion confirmed by recombinant ELISA and IHA in patients who were randomized and received at least one dose of nifurtimox in the 30-day treatment regimen; the proportion of responders who showed seronegative conversion by two types of assay and no evidence of established Chagas disease-related cardiomyopathy measured by ECG; and seroreduction (as reduction of ELISA OD) measured by recombinant ELISA and total purified antigen ELISA.

Exploratory analyses performed included the incidence rate of seroconversion by age at randomization according to the following age groups: ≤2 years, 3 to ≤6 years, 7 to ≤12 years, and 13 to <18 years.

Safety evaluations included the occurrence of AEs considered possibly related to nifurtimox treatment, AEs caused by protocol-required procedures, physical examination abnormalities, cardiac assessment by 12-lead ECG, and assessment of vital signs.

### Follow-up assessments.

At annual follow-up visits, patients underwent a physical examination, including measurements of height, weight, and vital signs. Body mass index, treatment-related AEs, and AEs caused by procedures required by the protocol were also recorded. In addition, a standard 12-lead ECG was performed at the discretion of the investigator, and details of any concomitant medications were recorded. Intermediate follow-up visits were conducted by telephone approximately 6 months after the 2- and 3-year follow-up visits to identify any potential clinical symptoms of Chagas disease. During these two intermediate visits, qualified staff interviewed patients and/or their legally authorized parents or representatives. In case of suspected findings, an unscheduled visit to the research site was requested to initiate appropriate diagnostic evaluations.

### Serological tests.

Blood samples for T. cruzi serological tests (recombinant ELISA [Chagatest ELISA recombinante; Weiner Lab, Rosario, Argentina], total purified antigen ELISA [Chagatest ELISA lisado; Weiner Lab], IHA [Chagatest HAI; Weiner Lab]) were taken at the annual follow-up visits. Depending on the participant's age, and at the researcher's discretion, these samples were collected under fasting conditions.

### Parasitological tests.

Blood was also collected for qPCR tests to detect the presence of T. cruzi DNA. The qPCR assay used duplex real-time PCR targeting T. cruzi satellite DNA and an internal amplification control as previously described (43).

### Statistical analyses.

In the primary outcome analysis, the difference in the incidence rate of patients treated with nifurtimox with seronegative conversion (60-day regimen) and the estimated seronegative rate from a historical placebo control group was tested using a Poisson two-sided 95% exact CI. The historical control group comprised children 6 to 12 years of age and seropositive for T. cruzi who received placebo when participating in a 48-month, double-blind, randomized clinical trial in northwestern Argentina (19). The children had indeterminate-phase Chagas disease, which was most likely to have been contracted more than 6 years before beginning treatment. In this historical control group, seronegative conversion was detected in two patients by conventional serology but in none by IHA (19). Thus, none of the patients in the placebo group was considered to have seroconverted, based on the criteria used in this study. The patient-time at risk was 176 patient-years, and the incidence rate estimate was 0% (0 of 176). Superiority was confirmed if the lower limit of the 95% CI for the incidence rate of seronegative conversion for patients in the nifurtimox 60-day regimen group was higher than the rate in the historical placebo control group (i.e., higher than 0%). The same approach was used as an exploratory analysis to calculate the incidence rate of seroconversion and 2-sided 95% CI in patients who received at least one dose of nifurtimox in the smaller 30-day treatment regimen and in all nifurtimox-treated patients by age category.

Recombinant ELISA OD values and total purified antigen ELISA were analyzed descriptively. The changes from baseline were summarized to show any serial reductions in OD values. Baseline was defined as the OD values of the same ELISAs measured at visit 1. The number of patients who showed seronegative conversion and no evidence of established cardiomyopathy was calculated overall and by treatment regimen. Baseline, efficacy, and safety analyses were performed using the full analysis set, based on patient randomization and treatment.

### Data availability.

The authors confirm that all data underlying the findings are fully available without restriction. All data files are available from the ClinicalTrials.gov website (trial identifier: NCT02625974).
