# Supplementary material for: Efficacy and Safety of Nifurtimox in Pediatric Patients with Chagas Disease: Results at 4-Year Follow-Up in a Prospective, Historically Controlled Study (CHICO SECURE)
Source: Antimicrob Agents Chemother. 2023 Mar 28;67(4):e01193-22. doi: 10.1128/aac.01193-22 (PMC10112190; doi:10.1128/aac.01193-22)
Supplement: Supplemental file 1 — Supplemental material. Download aac.01193-22-s0001.docx, DOCX file, 0.01 MB [file aac.01193-22-s0001.docx]

**SUPPLEMENTAL MATERIAL**

**CHICO SECURE Study Group study site principal investigators**

Argentina

Jaime Altcheh MD, PhD. Parasitología, Hospital de Niños Ricardo Gutiérrez and Instituto Multidisciplinario de Investigacion en Patologias Pediatricas (IMIPP), CONICET-GCBA, Gallo 1330, C1425, Buenos Aires, Argentina

L. Arce MD, Hospital Pediátrico Dr. Fernando Barreyro, Mariano Moreno 110, 3300 Posadas, Argentina

M. Caruso MD, Hospital de Niños Dr. Héctor Quintana, José Hernández 624, 4600 San Salvador de Jujuy, Argentina

R. de la Fuente MD, Hospital Papa Francisco, Barrio Solidaridad, Etapa 4, Manzana 405B, 4400 Salta, Argentina

M. Díaz Ariza MD, Centro Integral Médico Actitud, B° Faldeo del Velasco Sur, Manzana D, Casa 3 La Rioja, Argentina

C. Domínguez MD, Hospital Lagomaggiore, Timoteo Gordillo S/N, 5500 Mendoza, Argentina

R. Duarte MD, Sanatorio San Juan, San Juan 975, W3400CBI Corrientes, Argentina

G. Ensinck MD, Hospial de Niños Victor J Vilela, Virasoro 1855, 2000 Rosario, Argentina

A. Falaschi MD, Hospital Pediátrico Dr. Humberto Notti, Av Bandera de Los Andes 2603, 5519 Mendoza, Argentina

M. Jofre MD, Vacunatorio Provincial San Juan, Bartolomé Mitre 344 Oeste, 5400 San Juan, Argentina

C. Llapur MD, Hospital de Clínicas Presidente Dr. Nicolás Avellaneda, Catamarca 2000, 4000 Tucumán, Argentina

I. Menna MD, Hospital Fernández, Cerviño 3354, C1425AGP Buenos Aires, Argentina

C. Monla MD, Hospital Público Materno Infantil, Av Sarmiento 1301, 4400 Salta, Argentina

G. Moscatelli MD, Hospital de Niños Ricardo Gutiérrez, Gallo 1330, C1425EFD Buenos Aires, Argentina

J. Morales MD, Hospital de Niños Sor María Ludovica, Calle 14 no 1631 entre 65 y 66, 1900 La Plata, Argentina

T. Ramirez MD, Centro de Enfermedad de Chagas y Patologias Regionales, Av. Belgrano Norte 660, Santiago del Estero, Argentina

A. Romano MD, Instituto de Diagnóstico e Investigaciones Médicas de Formos, Jujuy 572, 3600 Formosa, Argentina

M. Sosa MD, Hospital de Enfermedades Infecciosas Dr. F. J. Muñiz, Av Vélez Sarsfield 405, 1281 Buenos Aires, Argentina

Colombia

J. Dib MD, Centro Ensayos Clínicos - Fundación Salud para el Trópico, Km 21 Troncal del Caribe, 0 Santa Marta, Colombia

J. Dib MD, Fundación Hospital Universidad del Norte, Calle 30 Autopista al Aeropuerto al lado del parque Muvdi- Soledad/Atlantico, Barranquilla, Colombia

V. Sierra MD, Centro de Atención e Investigación Médica CAIMED Yopal, Calle 17 No. 26 05, Los Helechos, 0 Yopal, Colombia

Bolivia

L. Ortiz Daza MD, Universidad Autónoma Juan Misael Saracho - Plataforma de Chagas – Tarija, España esquina Pasaje California, El Tejar, Tarija, Bolivia

J. Pinto MD, Fundación CEADES Plataforma de Chagas Cochabamba, Av. Aniceto Arce y Oquendo, Cochabamba, Bolivia

J. Sanchez MD, Fundación CEADES Hospital Manuel Ascencio Villarroel de Punata, Calle Gral. Achá 632 Punata, Bolivia
